# The Finding of Posterior Wall Low-Voltage Zones During Cryoballoon Pulmonary Vein Isolation Facilitated by Periprocedural Electroanatomical Mapping Is Associated with a Worse Ablation Outcome

**DOI:** 10.3390/jcdd13060287

**Published:** 2026-06-22

**Authors:** Maxime Tijskens, Benjamin De Becker, Michael Wolf, Bruno Schwagten, Yves De Greef

**Affiliations:** 1 Department of Cardiology, ZAS Heart Centre Middelheim, 2020 Antwerp, Belgium; benjamin.debecker@zas.be (B.D.B.); michael.wolf@zas.be (M.W.); bruno.schwagten@zas.be (B.S.); yves.degreef@zas.be (Y.D.G.); 2 Department of Cardiology, AZ Rivierenland Hospital, 2880 Bornem, Belgium; 3 Heart Rhythm Management Centre, European Reference Networks Guard-Heart, Universitair Ziekenhuis Brussel Heart Rhythm Research Brussels, Postgraduate Program in Cardiac Electrophysiology and Pacing, Vrije Universiteit Brussel, 1090 Brussels, Belgium

**Keywords:** atrial fibrillation, cryoballoon ablation, electroanatomical mapping, low-voltage zones, posterior wall, recurrence

## Abstract

Background: The presence of left atrial fibrosis is a marker of advanced remodeling and is associated with a worse outcome after pulmonary vein isolation (PVI). Conventional fluoroscopy-only cryoballoon ablation (CBA) lacks this prognostic information. The addition of electroanatomical mapping (EAM) using the inner lumen spiral catheter allows accurate voltage assessment of the left atrial posterior wall. However, the value of the finding of posterior wall low-voltage zones (pwLVZs) is unknown. Purpose: To study the value of left atrial voltage maps during CBA by comparing clinical and procedural characteristics and clinical outcome between patients with and without pwLVZs. Methods: A cohort of 250 consecutive patients who underwent index CBA for atrial fibrillation was analyzed. All patients underwent pre- and post-procedural EAM using the Achieve^TM^ catheter and EnSite^TM^ mapping system. The presence of LVZs was evaluated at the postprocedural voltage map of the posterior wall. Clinical success was defined as freedom from documented AF or atrial tachycardia (AT) >30 s after 1 year. Results: PwLVZs were found in 41/250 (16.4%) of patients. Patients with pwLVZs were older (69.3 ± 8.5 vs. 64.2 ± 10.4; *p* = 0.003), more frequently female (63.4% vs. 32.5%; *p* < 0.001) and had higher CHA_2_DS_2_-VASc scores (3.0 ± 1.6 vs. 2.0 ± 1.5; *p* < 0.001). The incidence of obesity (31.7% vs. 25.8%; *p* = 0.048), structural heart disease (35.5% vs. 17.4%; *p* = 0.021) and persistent AF (68.3% vs. 43.8%; *p* = 0.004) was higher in the pwLVZs group. Kaplan–Meier analysis of clinical outcome showed a higher recurrence rate in the pwLVZs group. The finding of pwLVZs was a predictor of atrial arrhythmia recurrence during follow-up (HR 2.583; 95%CI: 1.334–5.002; *p* = 0.005). Conclusions: In CBA facilitated by integrated EAM, pwLVZ was associated with older age, female sex, higher CHADS-VASc scores, obesity, structural heart disease and persistent AF. The finding of pwLVZs is predictive of a worse clinical outcome.

## 1. Introduction

Atrial fibrosis is a major determinant in the development and progression of AF [1]. The presence of atrial low-voltage zones (LVZs) in high-density electroanatomical mapping (EAM) is considered a surrogate marker for atrial fibrosis [2]. The finding of LVZs during an ablation procedure for atrial fibrillation (AF) is an independent predictor of recurrence after radiofrequency ablation (RFA) [3,4,5]. Because of the absence of mapping, this prognostic information is not available in conventional fluoroscopy-only cryoballoon ablation (CBA). However, in our center, we have a long tradition of CBA facilitated with low-density EAM using an inner lumen spiral catheter, with the advantage of improved acute pulmonary vein isolation (PVI) rate and 1-year clinical outcome [6,7]. This integrated approach allows accurate assessment of the left atrial posterior wall and assessment of LVZs, without the need for a separate mapping catheter. The goal of the current study was to assess whether the finding of LVZs at the level of the posterior wall of the left atrium (LA) is associated with certain clinical characteristics and has a prognostic value.

## 2. Methods

### 2.1. Study Population

In this observational, retrospective single-center study, we analyzed 250 consecutive AF patients who underwent a first CBA between January and September 2020 at the ZAS Middelheim Heart Center in Antwerp. Patients with previous PVI or cardiac surgery were excluded from the study. The study was approved by the local ethics committee.

### 2.2. Cryoballoon Ablation Procedure

The setup of our CBA protocol has been previously described in detail [8]. In brief, after gaining access to the LA by fluoroscopy- and transesophageal echocardiography-guided transseptal puncture, a 15-Fr steerable sheath (Flex Cath 18 Advance^TM^, Medtronic Inc., Minneapolis, MN, USA) was advanced into the LA cavity, followed by a 28 mm CB (Arctic Front Advance^TM^ or Arctic Front Advance Pro^TM^, Medtronic Inc., Minneapolis, MN, USA) with a octapolar 20 mm diameter inner lumen spiral mapping catheter (Achieve^TM^, Medtronic Inc., Minneapolis, MN, USA). Then the CB was advanced over the Achieve, inflated in the LA, and positioned at each PV ostium. Optimal vessel occlusion was confirmed by selective PV angiograms. Prior to ablation, all efforts were made to record LA-PV potentials with the Achieve catheter (moving to a more proximal position, different torquing movements, etc.), allowing real-time monitoring of PV entrance block. Veins were ablated using a single freeze strategy per vein, either 180 or 240 s. The choice for either of them was left to the operator’s discretion. However, if at 60 s freeze time, pulmonary vein potential (PVP) did not disappear, and/or the temperature of −40 °C was not reached, cryoablation was either aborted and followed by catheter repositioning to attempt a better occlusion, or continued, and a second application was given in order to reach the target parameters mentioned above. The right phrenic nerve (PN) was monitored continuously during the ablation of the right-sided PVs. In case of transient phrenic nerve injury, no additional cryo-ablations were applied at the level of the right PVs. Only PVI was the goal in all cases, without further substrate ablation (no posterior wall isolation). At the end of ablation, all PVs were re-checked in sinus rhythm with the Achieve catheter to confirm PVI defined as PV entrance block. Pacing maneuvers were used to distinguish residual PVPs from far-field signals (pacing from superior vena cava and distal coronary sinus for right-sided and left-sided pulmonary veins, respectively).

### 2.3. Perprocedural Electroanatomical Mapping

**Figure 1:**
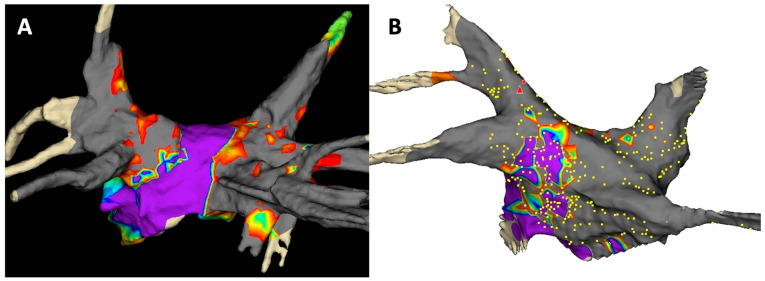
Representative posterior wall voltage maps (after PVI) in 2 different patients. Panel (**A**): Normal voltages at the posterior wall. Panel (**B**): Posterior wall low-voltage zones. Yellow dots are given as a representation of the mapping density.

Integrated EAM was performed using the Achieve catheter (inner lumen circular mapping catheter) and EnSite^TM^ cardiac mapping system (Abbott Inc., St. Paul, MN, USA). LA voltage maps specifically mapping in detail the pulmonary veins (PVs), PV antral region and posterior wall were performed pre- and post-CBA. The former was used to define anatomy and to guide ablation, while the latter was used for PVI validation and to assess the presence of LVZs. LVZs were only evaluated at the posterior wall, since inadequate wall contact in other zones might lead to “pseudo LVZs”. EAM was performed in sinus rhythm whenever possible. Patients in AF at the start of the procedure underwent external direct current cardioversion, and this was repeated when necessary to be able to evaluate the presence of pwLVZs on postprocedural EAM in sinus rhythm. PwLVZs were defined using the conventional cutoff of <0.5 mV, as reported in previous RFA studies [4,9]. Representative posterior wall voltage maps showing pwLVZs are depicted in Figure 1.

### 2.4. Post-Procedural Management and Follow-Up

After the procedure, subcutaneous low-molecular-weight heparin was administered to all patients, as well as oral anticoagulation therapy, either a vitamin K antagonist (target INR between 2.0 and 3.0) or a direct oral anticoagulant. Antiarrhythmic drug treatment was reinstituted in all patients. After 3 months, oral anticoagulation therapy was continued except in the case of a CHA_2_DS_2_-VASc score of 0. All antiarrhythmic drugs were invariably stopped at the latest after 3 months.

All patients underwent conventional follow-up with questionnaire, physical examination and electrocardiogram at scheduled visits (at 2, 6 and 12 months in the first year and at least yearly thereafter) and at unscheduled visits (if symptomatic). In the latter, the related arrhythmia was documented either by ECG, Holter monitoring (1 to 7 days), or event recording. When patients had a cardiac implantable electrical device, device interrogation was also used to confirm arrhythmia recurrence.

The primary endpoint for this analysis was clinical success, defined as freedom from documented atrial arrhythmia (AF or atrial tachycardia) at least 30 s in duration after a single first procedure without anti-arrhythmic drugs, considering a blanking period of 2 months, as recommended by the Expert Consensus Statement [10].

### 2.5. Repeat Ablation Procedure

All repeat ablations were performed using RF energy. After double transseptal puncture, a 3D electro-anatomical map of the LA was acquired. A 3-dimensional (3D) reconstruction of the LA was made guided by a 3D non-fluoroscopic navigation system (CARTO^TM^, Biosense-Webster, Johnson & Johnson, Irvine, CA, USA) and a dedicated mapping catheter (Pentaray^TM^ or Octaray^TM^, Biosense Webster, Johnson & Johnson, Irvine, CA, USA). RF ablation was performed with an open-irrigated-tip catheter with contact-force monitoring (Thermocool Smarttouch^TM^, Biosense Webster, Johnson & Johnson, Irvine, CA, USA) in power-controlled mode with a power limit of 45 W and maximum temperature of 48 °C targeting an ablation index of 400 at the posterior wall and 550 at the anterior wall. The power was reduced at the posterior wall to 30 W and further adjusted in case of esophageal temperature rise during ablation.

If PVI proved to be durable at baseline, a further ablation strategy was performed on a case-by-case basis at the discretion of the operator. The ablation strategy was categorized as targeted or empirical ablation. In targeted ablation, an induced non-PV trigger or atrial arrhythmia was targeted. In empirical ablation, common non-PV triggers or substrates (mainly LVZs) were targeted. When both targeted and empirical targets were ablated, the ablation strategy was considered targeted.

### 2.6. Statistical Analysis

Categorical variables are expressed as absolute and relative frequencies. The Shapiro–Wilk test was used to examine if a continuous variable was normally distributed. Continuous variables are expressed as mean ± standard deviation or median and range as appropriate. Comparisons of continuous variables were done with a Student’s *t*-test or Mann–Whitney U test as appropriate, and comparisons of categorical variables with the χ^2^ or Fisher’s exact test as appropriate. Event-free survival rates were estimated by the method of Kaplan–Meier. The log-rank test was used to detect significant differences between groups. To assess the contribution of baseline patient characteristics to the recurrence of arrhythmias, multivariable Cox proportional hazard regression analysis was used. Only variables that were statistically significant (*p* < 0.05) in univariable analysis were used for the multivariable analysis. Statistical analyses were conducted using SPSS data-analytical software (SPSS v24, Chicago, IL, USA).

## 3. Results

### 3.1. Clinical Characteristics

Of the 250 patients included in the study, 41 (16.4%) had pwLVZ, as defined in the Methods section. In the study cohort, patients were predominantly male (61.3%) with a mean age of 65.0 ± 10.3 and a CHAD-VASc score of 2.1 ± 1.5. Persistent AF was present in 47.6% and structural heart disease in 15.8%. On average, LA diameter was 39.2 ± 7.4.

The comparison of characteristics of patients with and without pwLVZ is listed in Table 1. Patients with pwLVZ were older (69.3 ± 8.5 vs. 64.2 ± 10.4; *p* = 0.003), more frequently female (63.4% vs. 32.5%; *p* < 0.001) and had higher CHA_2_DS_2_-VASc scores (3.0 ± 1.6 vs. 2.0 ± 1.5; *p* < 0.001). The incidence of obesity (31.7% vs. 25.8%; *p* = 0.048), structural heart disease (35.5% vs. 17.4%; *p* = 0.021) and persistent AF (68.3% vs. 43.8%; *p* = 0.004) was higher in the pwLVZ group.

There was no significant difference in LA diameters (40.6 ± 7.1 vs. 38.9 ± 7.5 mm; *p* = 0.260) and diagnosis-to-ablation time (16.5 ± 25.8 vs. 28.3 ± 42.1 months; *p* = 0.086) between groups.

### 3.2. Clinical Outcome and Predictors

Acute successful isolation of all PVs was reached in all patients. The mean follow-up was 23.9 ± 7.8 months for the total cohort with no difference between groups (21.6 ± 7.5 vs. 24.2 ± 7.9 months; *p* = 0.079). Recurrence of atrial arrhythmia after index CBA was significantly higher in patients with pwLVZ both after 1 year (12/41 (29.3%) vs. 21/209 (10.0%); *p* < 0.001) and during total follow-up (20/41 (48.8%) vs. 41/209 (19.6%); *p* < 0.001).

**Figure 2:**
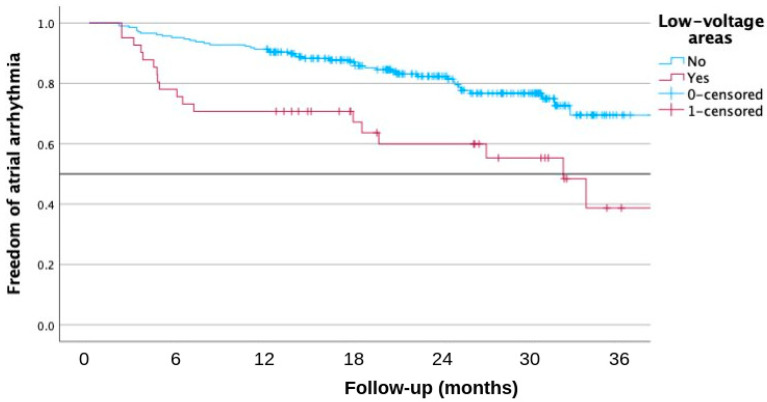
Kaplan–Meier curve comparing outcome after index ablation. *p* < 0.001.

Kaplan–Meier curves describing freedom from atrial arrhythmia during follow-up after one ablation (index CBA) in patients with and without pwLVZ are shown in Figure 2. Both curves diverge early, with the steepest difference occurring during the first 6 months of follow-up. In the subgroup of patients with a recurrence, the mean time to recurrence was not different between the two groups (13.4 ± 14.3 vs. 14.3 ± 8.9 months; *p* = 0.769).

The type of recurrent atrial arrhythmia was not significantly different between groups: 8/20 (40.0%) paroxysmal AF, 10/20 (50.0%) persistent AF and 2/20 (10.0%) atrial tachycardia in the pwLVZ group vs. 20/41 (48.8%) paroxysmal AF, 15/41 (36.6%) persistent AF and 6/41 (14.6%) atrial tachycardia in the no pwLVZ group (*p* = 0.596).

Univariable and multivariable adjusted predictors of atrial arrhythmia recurrence after CBA are outlined in Table 2. We identified both the presence of pwLVZ (HR 2.583; 95%CI: 1.334–5.002; *p* = 0.005) and LA size (HR 1.055; 95% CI (1.016–1.096); *p* = 0.006) as independent predictors.

### 3.3. Findings During Repeat Procedures

In the subgroup of 61/250 patients with a recurrence during follow-up (20/41 patients with pwLVZs and 41/209 without pwLVZs), 37 underwent a repeat procedure: 14/20 (70.0%) in the patients with pwLVZs and 23/41 (56.1%) in the patients without pwLVZs (*p* = 0.230). In this small subgroup, we did not find a significant difference in the proportion of patients who underwent a first repeat ablation within 1 year after index CBA (10/14 (71.4%) vs. 14/23 (60.9%); *p* = 0.514).

Reconnection of at least one pulmonary vein was present in 3/14 (21.4%) patients with pwLVZs at index CBA in comparison to 11/23 (47.8%) patients without posterior wall LVZs (*p* = 0.108). There was a higher number of patients treated with an empirical ablation strategy at repeat ablation in the cohort of patients with pwLVZs at index CBA (9/14 (64.4%) vs. 7/23 (30.4%); *p* = 0.044).

**Figure 3:**
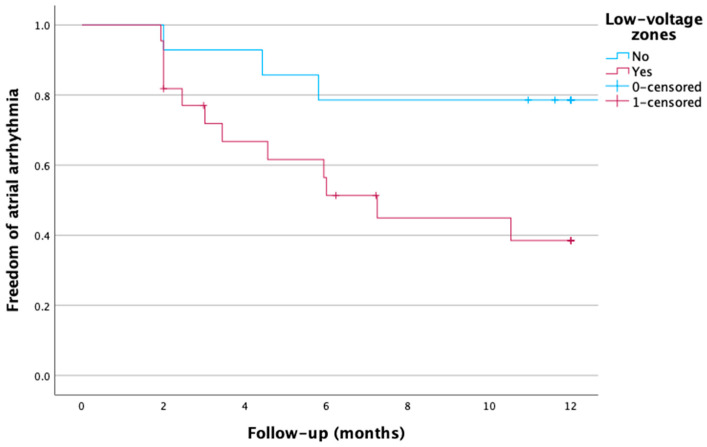
Kaplan–Meier curve comparing outcome after first repeat ablation. *p* = 0.034.

Kaplan–Meier curves describing freedom from atrial arrhythmia during follow-up after two ablations (index CBA + first repeat RF ablation) in patients with and without pwLVZs are shown in Figure 3. This showed a worse outcome in the patients with pwLVZs again at index CBA with a steep divergence of both curves during the first months after ablation.

## 4. Discussion

### 4.1. Main Findings

To the best of our knowledge, the current study is the first to evaluate the value of the finding of pwLVZs using low-density electroanatomical mapping during index CBA for AF. The main findings of our study are:

1. Patients with pwLVZs were older, more frequently female, had higher CHA_2_DS_2_-VASc scores and a higher incidence of obesity, structural heart disease and persistent AF.
2. Patients with pwLVZs had a worse outcome at 1 year after index ablation, and the presence of pwLVZs was a predictor of recurrence.
3. During repeat procedure, patients with pwLVZs were more likely to undergo empirical substrate ablation. Patients with pwLVZs at the index procedure had a worse outcome at 1 year after the first repeat ablation.

### 4.2. Addition of Electroanatomical Mapping to Cryoballoon Ablation

The addition of periprocedural EAM to CBA has been shown to improve acute PVI rate in comparison to conventional only fluoroscopy-guided CBA in AF patients [7]. We consider this superior acute PVI validation as the reason for a better 1-year clinical outcome of this approach [6]. It can also facilitate CBA in patients with challenging anatomies, such as dextrocardia [11]. The workflow using the Achieve catheter (already an integral part of the CBA system) as a mapping catheter in combination with the EnSite^TM^ cardiac mapping system limits costs and complexity by avoiding the time-consuming process of switching to a separate mapping catheter.

### 4.3. Posterior Wall Low-Voltage Zones

Of note, in the current study, we only evaluated LVZs at the posterior wall of the left atrium because of the concern that suboptimal wall contact in other zones might lead to “pseudo-LVZs”. This contrasts with previous studies regarding RFA procedures in which LVZs were described in different atrial zones by the use of high-definition EAM [2,4,12,13]. In our study, pwLVZs were found in 41/250 (16.4%) of patients. In previous studies, the incidence of posterior wall LVZs varied depending on the population. In the recent ERASE-AF trial, LVZs were reported in 36% of patients undergoing first radiofrequency ablation for persistent atrial fibrillation. Of those patients, 53% had LVZs located at the posterior wall. However, in the ERASE-AF trial, LVZs were more prevalent in different regions: anterior wall (75%) and septal (65%) [12].

In the current study, the finding of pwLVZs was associated with older age, female sex, higher CHADS-VASc scores, obesity, structural heart disease and persistent AF. Of interest, there was no correlation with left atrial diameter, another known predictor of recurrence after ablation. The complex interplay between AF risk factors and predictors of recurrence after ablation remains incompletely understood and warrants further research. In a recent paper, our group showed a higher prevalence of chronic PVI and LVZs leading to more empirical substrate ablation in women in a big cohort of repeat ablations after index CBA, suggesting a less PV- and more substrate-based AF pathophysiology in women [14]. In the MASH-AF II study, the hypothesis of two different “fibrotic pathways” in patients with AF was proposed. One pathway of first progressive atrial dilatation followed by extensive fibrosis was more prevalent in male patients with longer diagnosis-to-ablation time and more prevalent persistent AF. The other pathway of progressive fibrosis without preceding left atrial dilatation was more prevalent in female and older patients with higher CHADS-VASc scores. The authors suggested that in younger, especially male patients with significant left atrium dilatation, traditionally considered poor candidates for ablation, the procedure may have better-than-expected outcomes. On the contrary, in older, mainly female patients with higher CHADS-VASC scores, even without significant left atrium dilatation, extensive fibrosis may be present, and the outcome may be worse [2]. In the MASH-AF II study, high-density EAM was used during the RFA index procedure with extensive post hoc off-line quantitative measurements. On the contrary, in our study, only qualitative measurements using an ad hoc integrated non-high-density EAM of the posterior wall made it possible to differentiate between a better or worse outcome after index CBA.

### 4.4. Posterior Wall Low-Voltage Zones During Cryoballoon Ablation Predict Worse Clinical Outcome

The current study included a “real-world” cohort of AF patients scheduled for first AF ablation in a single center with vast experience in the addition of EAM to CBA. In this cohort, clinical success after a single procedure and 1 year of follow-up was 86,8%. However, clinical success was significantly lower in patients with pwLVZs after 1 year (29/41 (70.7%) vs. 188/209 (90.0%); *p* < 0.001).

The finding of LVZs using high-density EAM during an ablation procedure for AF is a long-known independent predictor of recurrence after radiofrequency ablation (RFA). Verma et al. reported already 20 years ago that the finding of LA LVZs using high-density mapping in AF patients undergoing first ablation using radiofrequency energy targeting isolation of PVs and superior vena cava predicted a worse clinical outcome (HR 3.4, 95%CI (1.3–9.4); *p* = 0.01). Schade et al. showed that the finding of LVZs was the only predictor of atrial arrhythmia recurrence after a voltage-guided ablation strategy in patients undergoing first radiofrequency ablation for persistent AF (HR 5.9, 95%CI (2.2–16.2); *p* < 0.001) [3,4,5,15]. Because of the absence of mapping, this information is not available in conventional fluoroscopy-only CBA. For the first time, the current study shows that the finding of pwLVZs using low-density EAM is independently associated with a worse clinical outcome (HR 2.583; 95%CI: 1.334–5.002; *p* = 0.005), without the need for an extra mapping catheter by solely using the inner lumen Achieve mapping catheter (only 8 poles). It is important to acknowledge that this association is observational and does not establish causality.

When pwLVZs are present, the posterior wall may serve as an autonomous source of non-PV triggers or reentrant circuits that sustain AF irrespective of the completeness of PVI. Regarding the relationship between AF type and posterior wall substrate, the literature consistently demonstrates a strong association between non-paroxysmal AF and the prevalence and extent of LVZs. In a large cohort of 539 patients, Huo et al. reported LVZs in 18.8% of paroxysmal AF patients compared to 54.3% of patients with persistent AF (*p* < 0.001), with LVZ areas significantly larger in the persistent AF group across all left atrial regions, including the posterior wall [12]. These findings are consistent with the concept that AF perpetuation itself drives progressive atrial structural remodeling. In the current study, despite a high prevalence of persistent AF in the pwLVZ group (68.3% vs. 43.8%), pwLVZ remained an independent predictor of recurrence after adjustment for AF type, suggesting that posterior wall substrate carries additional prognostic information beyond AF type alone.

### 4.5. Clinical Implications and Future Perspectives

The finding of pwLVZ during index CBA permits the electrophysiologist to predict a higher chance of atrial arrhythmia recurrence during follow-up and to inform the patient accordingly. In the small exploratory subgroup of 37 patients undergoing repeat ablation, those with pwLVZ at the index procedure tended to have worse outcomes. However, given the limited sample size and heterogeneous procedural strategies, these findings should be considered hypothesis-generating only and do not allow conclusions regarding optimal repeat ablation strategy in this population. Further studies have to investigate whether the best therapeutic strategy in these patients consists of repeat ablation or stringent control of comorbidities, and/or an ablate and pace strategy might be considered.

Posterior wall isolation in addition to PVI during the first ablation procedure has been attempted to improve clinical outcome, mainly in patients with persistent AF. However, so far, no clear clinical benefit has been shown using different energy sources, except when cryo-energy was used [16,17,18,19]. The ERASE-AF trial showed a better clinical outcome in patients with symptomatic persistent AF undergoing first RFA and individualized substrate ablation targeting low-voltage zones compared to PVI alone using radiofrequency energy [12]. Comparably, the addition of electroanatomical mapping to CBA might make it possible to identify patients benefiting from posterior wall isolation and to facilitate this additional ablation.

The addition of electroanatomical mapping also holds promise in emerging technologies such as pulsed field ablation. The importance of substrate identification in non-paroxysmal AF is further underscored by recent advances in pulsed field ablation (PFA). Della Rocca et al. described a structured multielectrode PFA-based strategy for persistent and long-standing AF, incorporating posterior wall isolation and electrogram-guided substrate ablation as integral components of the ablation protocol, achieving AF termination in 95.8% of cases and a single-procedure success rate of 74.6% at 15 months of follow-up [20]. Notably, low-voltage electrogram identification using the multielectrode PFA catheter was central to guiding substrate-based ablation in that study, analogous to the posterior wall voltage assessment described in the present study using the Achieve catheter during CBA. As PFA becomes increasingly adopted worldwide, the integration of periprocedural electroanatomical mapping for substrate characterization may provide a generalizable workflow to identify patients who require ablation beyond PVI, irrespective of the energy source employed.

### 4.6. Limitations

The main strength of the current study is the consecutive inclusion of patients in a large-volume center. However, some limitations should be acknowledged. First, this study is retrospective and single-center, which may restrict our ability to draw substantial conclusions. Second, only a small subgroup of patients had cardiac implantable electronic devices and therefore asymptomatic atrial arrhythmia recurrence might have occurred unnoticed, leading to an overestimation of the ablation success rate. Third, due to concerns of pseudo-LVZs as a result of suboptimal wall contact by the Achieve catheter in other LA zones, only pwLVZs were evaluated. Therefore, the incidence of LVZs in other regions and their impact on clinical outcomes could not be evaluated. However, the presence of LVZs at the level of the posterior wall only was found to be a predictor of worse clinical outcome. Voltage maps using our technique are less detailed than high-density voltage maps using dedicated mapping catheters, but the binary variable of the presence of pwLVZs was clinically relevant nevertheless. Fourth, the redo ablation subgroup comprised only 37 patients with heterogeneous procedural strategies at operator discretion, limiting the interpretability of the repeat procedure findings; these should be regarded as exploratory. Finally, formal interobserver agreement analysis and direct comparison with high-density mapping or MRI are important limitations to acknowledge, and future research is warranted.

## 5. Conclusions

The finding of pwLVZs using periprocedural EAM during index CBA is associated with a worse ablation outcome. This provides an additional advantage of the addition of periprocedural EAM to CBA.

## Figures and Tables

**Table 1:** Clinical characteristics.

	No pwLVZ (N = 209)	pwLVZ (N = 41)	*p*-Value
**Age**	64.2 ± 10.4	69.3 ± 8.5	**0.003**
**Female, n (**%)	68 (32.5%)	26/41 (63.4%)	**<0.001**
**Obesity**	54 (25.8%)	13/41 (31.7%)	**0.048**
**Arterial hypertension**	109 (52.4%)	23/41 (56.1%)	0.665
**Diabetes**	20/209 (9.7%)	6/41 (14.6%)	0.348
**History of stroke**	17/209 (8.2%)	3/41 (7.3%)	0.847
**CHADS-VASc score**	2.0 ± 1.5	3.0 ± 1.6	**<0.001**
**Sleep apnea**	16/209 (7.7%)	2/41 (4.9%)	0.525
**Structural heart disease**	29/209 (17.4%)	11/41 (35.5%)	**0.021**
**Left atrial diameter (mm)**	38.9 ± 7.5	40.6 ± 7.1	0.260
**Diagnosis-to-ablation time (months)**	28.3 ± 42.1	16.5 ± 25.8	0.086
**Persistent AF**	91/209 (43.8%)	28/41 (68.3%)	**0.004**

**Table 2:** Univariable and multivariable analysis of factors associated with AF/AFl/AT recurrence during follow-up.

	Univariate Analysis			Multivariate Analysis		
	HR	95% CI	*p*-value	HR	95% CI	*p*-value
**Persistent AF**	1.288	0.774–2.144	0.331			
**Diagnosis-to-ablation time**	1.004	0.998–1.009	0.158			
**CHADS-VASc score**	1.258	1.084–1.461	**0.003**	1.160	0.973–1.382	0.098
**Obesity**	0.862	0.480–1.547	0.618			
**Left atrial diameter**	1.063	1.023–1.105	**0.002**	1.054	1.014–1.097	**0.008**
**Structural heart disease**	1.257	0.637–2.483	0.510			
**Posterior wall LVZs**	2.520	1.448–4.386	**0.001**	2.583	1.334–5.002	**0.005**

## Data Availability

Data can be provided upon request.
